# Complete mitochondrial genome of *Cylindromyia* (*Calocyptera*) *intermedia* from Guiyang, China, and phylogeny of Phasiinae (Diptera: Tachinidae)

**DOI:** 10.1080/23802359.2022.2080021

**Published:** 2022-06-14

**Authors:** Rong Wang, Yan Zhi, Chuntian Zhang, Ming Yang, Jiayu Liu

**Affiliations:** a Key Laboratory of Medical Insects, Guizhou Medical University, Guiyang, China; b School of Basic Medical Science, Guizhou Medical University, Guiyang, China; c Laboratory Animal Center, Guizhou Medical University, Guiyang, China; d College of Life Sciences, Shenyang Normal University, Shenyang, China

**Keywords:** *Cylindromyia* (*Calocyptera*) *intermedia*, mitochondrial genome, phylogeny, Tachinidae

## Abstract

The mitochondrial genome of *Cylindromyia* (*Calocyptera*) *intermedia* (Meigen, 1824) was sequenced and assembled through high-throughput sequencing techniques and reference-based assembly methods. The mitochondrial genome is 15,114 bp in total, consisting of 13 protein-coding genes, 22 transfer RNA genes, 2 ribosomal RNA genes, and 1 noncoding control region. The nucleotide composition biases toward A and T; the overall A + T content are up to 76.9% of the entire mitogenome. The result of phylogenetic analysis suggested a close relationship between *C. intermedia* and subfamily Dexiinae.

Phasiinae is one of the four subfamilies of Tachinidae, however, there are only three mitochondrial genomes of this subfamily have been reported: *Ectophasia rotundiventris* (Loew, 1858), *Gymnosoma dolycoridis* (Dupuis, 1960) and *Subclytia rotundiventris* (Fallén, 1820) (Li et al. 2017; Pei et al. 2019). The genus *Cylindromyia* of tribe Cylindromyiini of subfamily Phasiinae includes 10 subgenera and 116 species worldwide, meanwhile 15 species are found in China (O’Hara et al. 2020). In this study, we sequenced the complete mitochondrial genome of *Cylindromyia* (*Calocyptera*) *intermedia* and analyzed its phylogenetic status, which provides useful genetic information for improving the taxonomic system and phylogenetics of Tachinidae.

The specimen (*C. intermedia*) was collected by sweeping collection from Huaxi District, Guiyang city, Guizhou Province, China (106.620159 N, 26.36755 E) on 7 June 2020 and was deposited at the Key Laboratory of Medical Insects of Guizhou Medical University (https://www.gmc.edu.cn/, Jiayu Liu, fsliujiayu@163.com) under the voucher number CI20200707. Total DNA was extracted from thoracic muscle tissues using Rapid Animal Genomic DNA Isolation Kit (Sangon Biotech Co., Ltd., Shanghai, China). The sequencing library was established and further sequenced using the Illumina Hiseq PE150 platform, followed by additional bioinformatic analyses outlined below. The initial annotation of the mitogenome, including gene prediction and non-coding RNA, was conducted using MITOS Web Server (http://mitos2.bioinf.uni-leipzig.de/index.py) (Bernt et al. 2013). Geneious Prime 2020.2.2 (Kearse et al. 2012) was used to compare the homologous gene annotations of the other two species of Phasiinae and then submitted data to GenBank database through NCBI.

The complete mitogenome of *C. intermedia* (GenBank accession number: OL539555) is 15,114 bp in length, with the following base composition: A (41%), T (35.9%), G (9%), and C (14.1%). It shows a conserved arrangement pattern, including 13 protein-coding genes (PCGs), two rRNA genes, 22 tRNA genes and one non-coding region. Four PCGs, two rRNA genes and eight tRNA genes are distributed in the light strand among the 38 sequence elements, while others are distributed in the heavy strand. The location and direction of all genes are conserved in respect to the other Phasiinae mitogenomes.

The 13 PCGs accounted for 73.9% of the complete mitogenome of *C. intermedia* (11,180 bp). PCGs utilize a variety of start codons including the standard ATN, except for the nonstandard CGA (*COI*) and TTG (*ND1*). The most frequent start codon was ATG, which was covered six PCGs (*COII*, *ATP6*, *COIII*, *ND4*, *ND4L*, *CYTB*). Nine PCGs (*ND2*, *COI*, *COII*, *ATP8*, *ATP6*, *COIII, ND4L*, *ND6*, *CYTB*) stop with TAA codon. *ND3* and *ND1* terminate with the codon TAG, while *ND5* and *ND4* have an incomplete stop codon T.

To investigate the phylogenetic status of *C. intermedia* in Phasiinae, a phylogenetic tree was reconstructed by the maximum-likelihood method using MEGAX 10.2.6 with bootstrap set to 1000 (Kumar et al. 2018) based on the combined 13 PCGs dataset for 17 tachinid species. *Lucilia sericata* (Meigen, 1826) from Calliphoridae and *Sarcophaga crassipalpis* Macquart, 1839 from Sarcophagidae were set as outgroups. The subfamily Phasiinae received a molecular phylogenetic treatment by Stireman et al. (2019, Fig. 4), the clade tribe Cylindromyiini of Phasiinae sometimes joins the subfamily Dexiinae in individual gene-trees. Our phylogenetic analysis showed a close relationship between *C*. *intermedia* of Phasiinae and *Rutilia goerlingiana* of Dexiinae (Figure 1), which was partially consistent with the previous work of Stireman et al. (2019). The complete mitogenome of *C*. *intermedia* will contribute to the further studies on molecular bases for the classification and phylogeny of between Phasiinae and other three subfamilies within Tachinidae.

**Figure 1.**
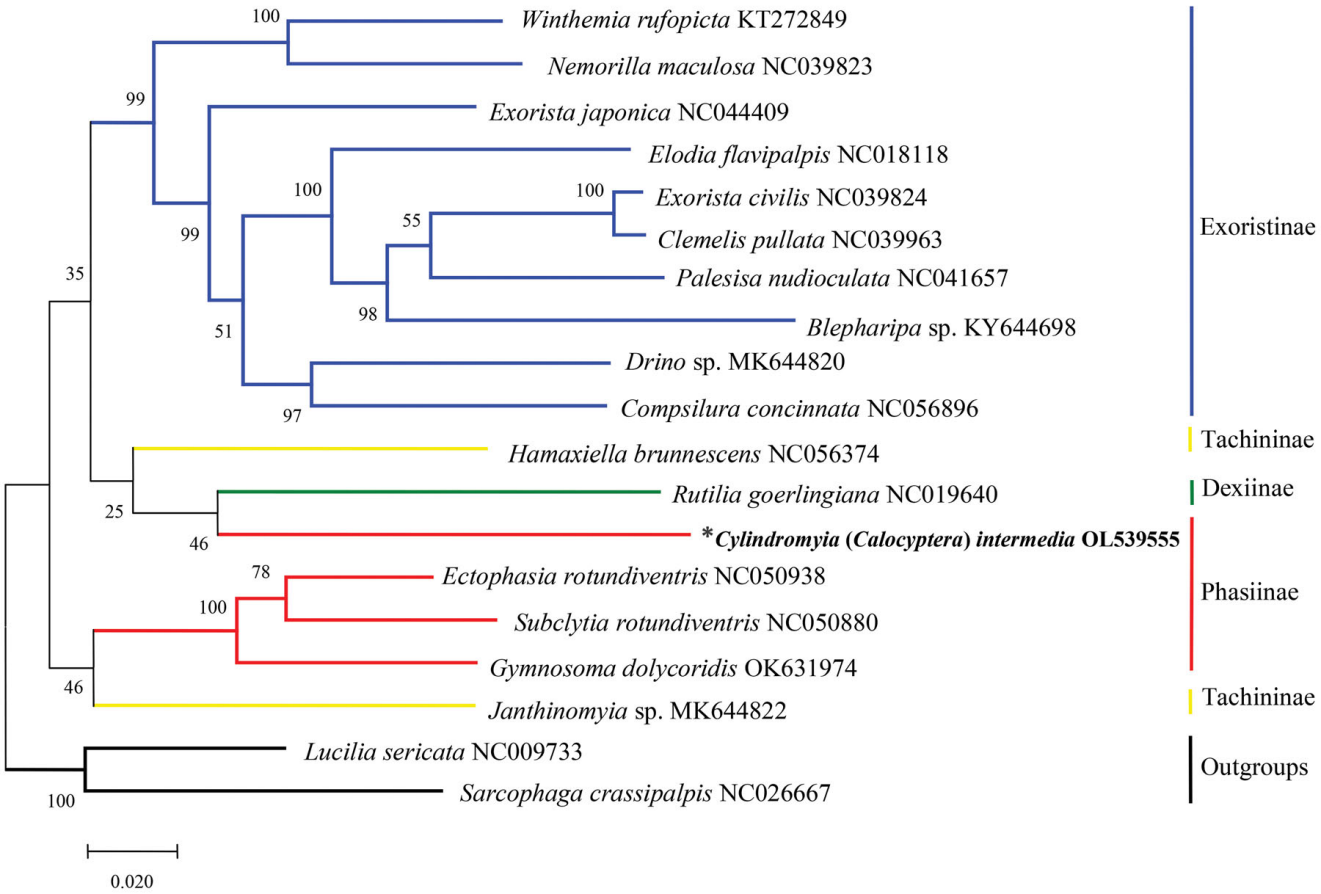
The maximum-likelihood phylogenetic analysis of 17 tachinid species based on the combined 13 PCGs. Accession numbers of mitochondrial sequences used in the phylogenetic analysis are listed after scientific name. *Species documented in this study.

## Ethical approval statement

The ethical approval (No. 1900074) was granted by the Animal Care Welfare Committee of Guizhou Medical University for the study.

## Author contributions

Ming Yang and Jiayu Liu were involved in the conception and design; Rong Wang and Jiayu Liu analyzed and interpreted the data; Rong Wang drafted the article; Yan Zhi and Chuntian Zhang revised it critically for intellectual content. All authors approved the final version to be published and agreed to be accountable for all aspects of the work.

## Data Availability

The genome sequence data that support the findings of this study are openly available in GenBank of NCBI at under the accession no. OL539555. The associated BioProject, SRA, and Bio-Sample numbers are PRJNA781126, SRR16980901, and SAMN23239677, respectively.
